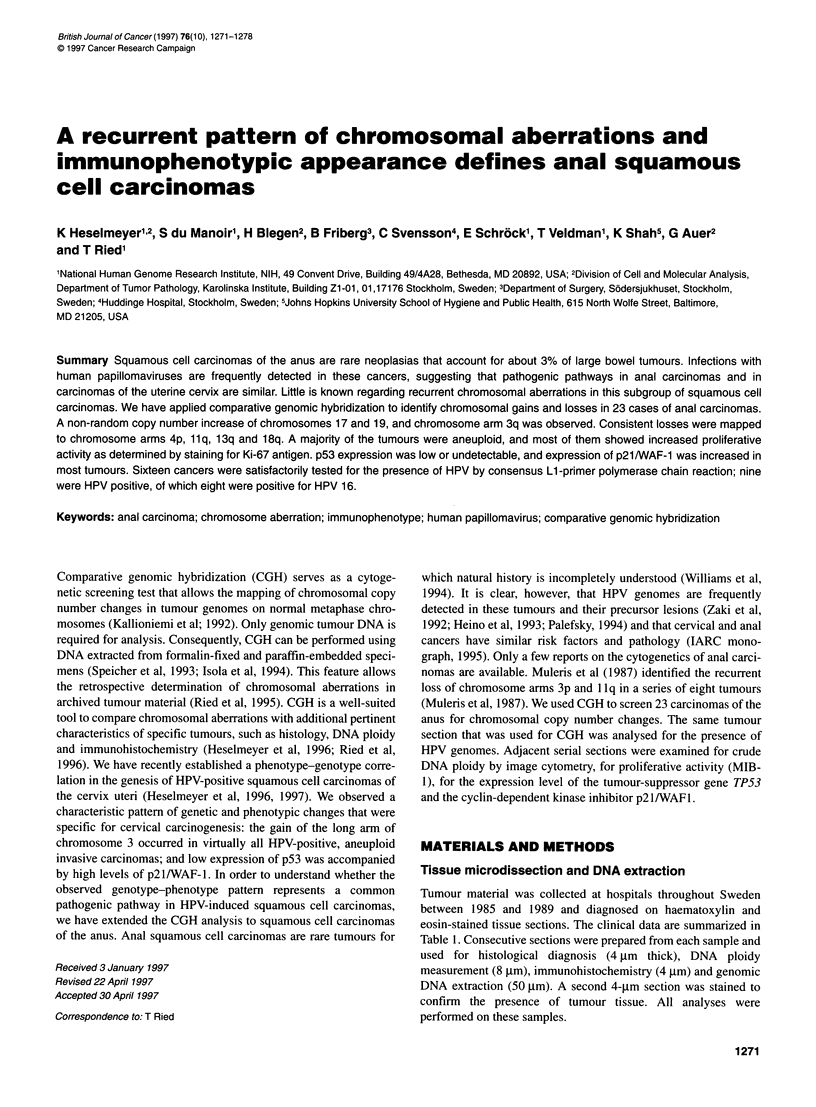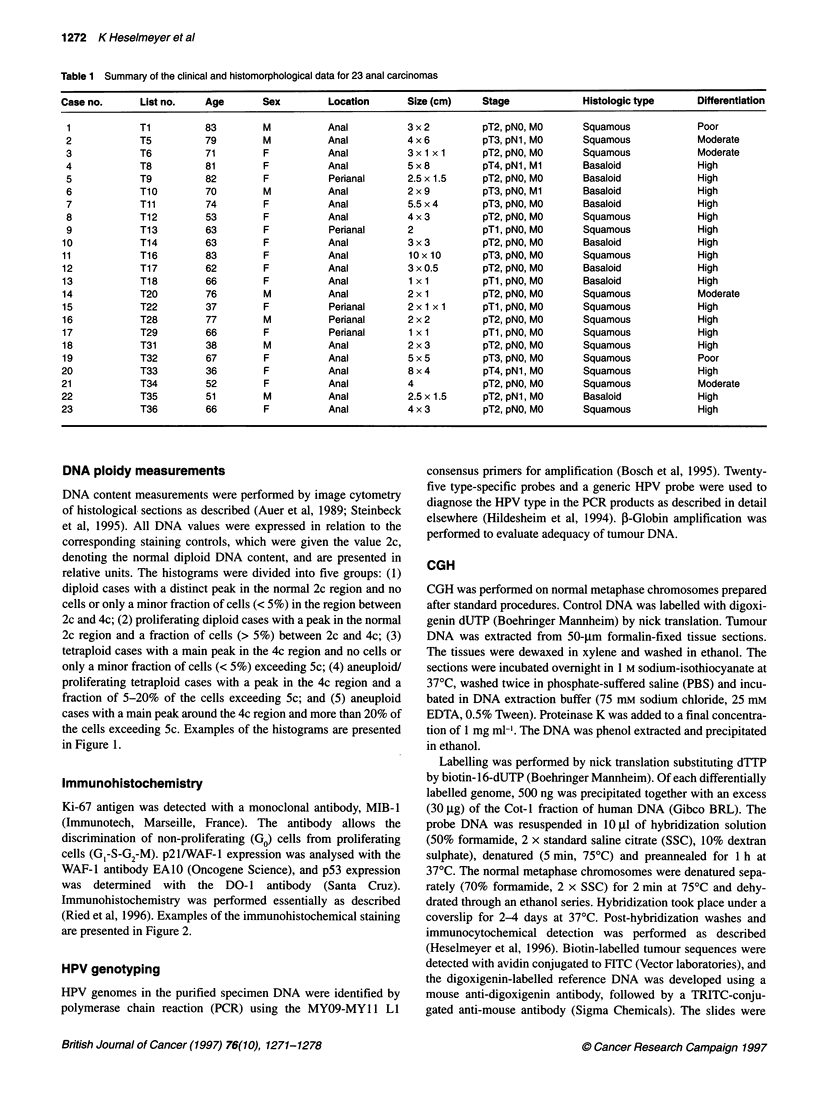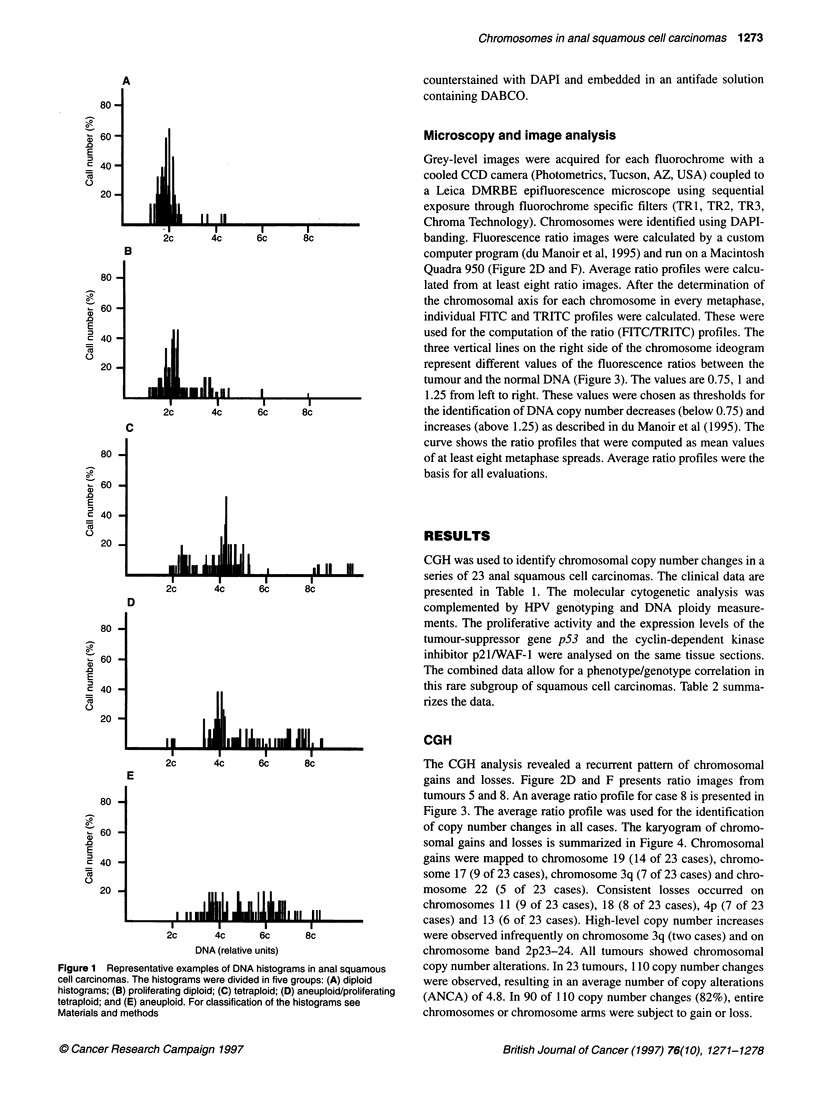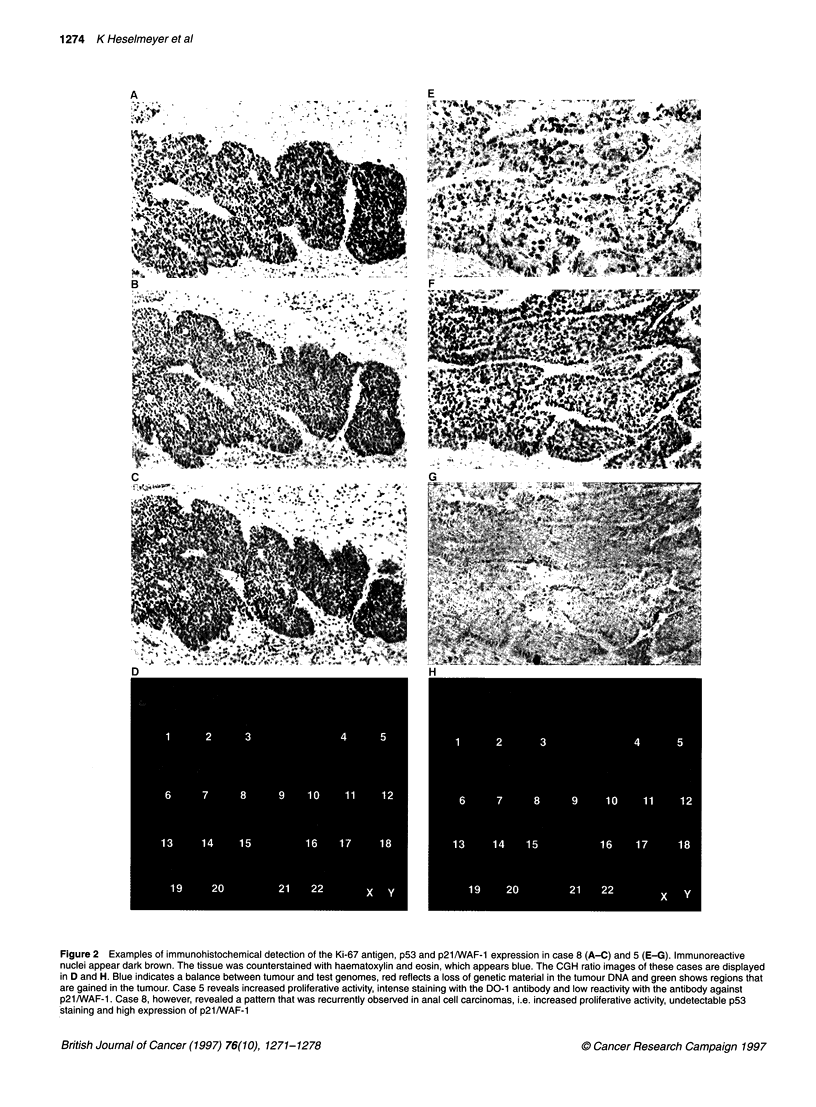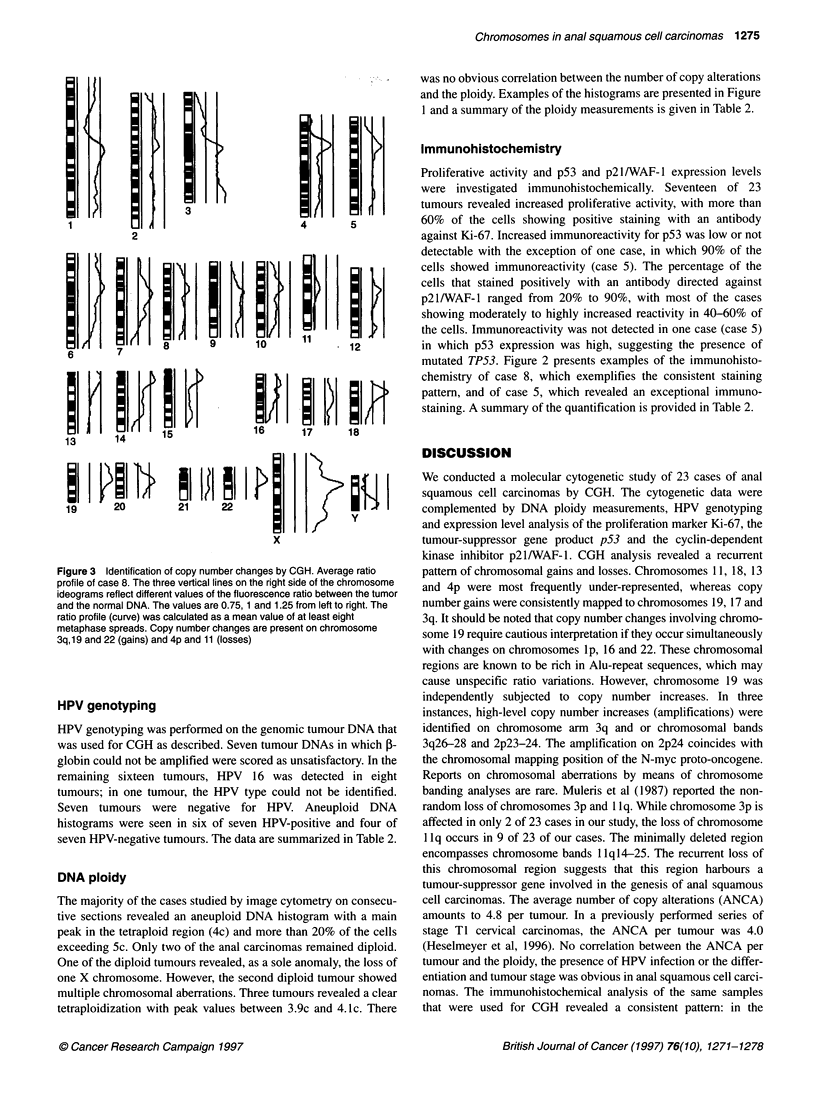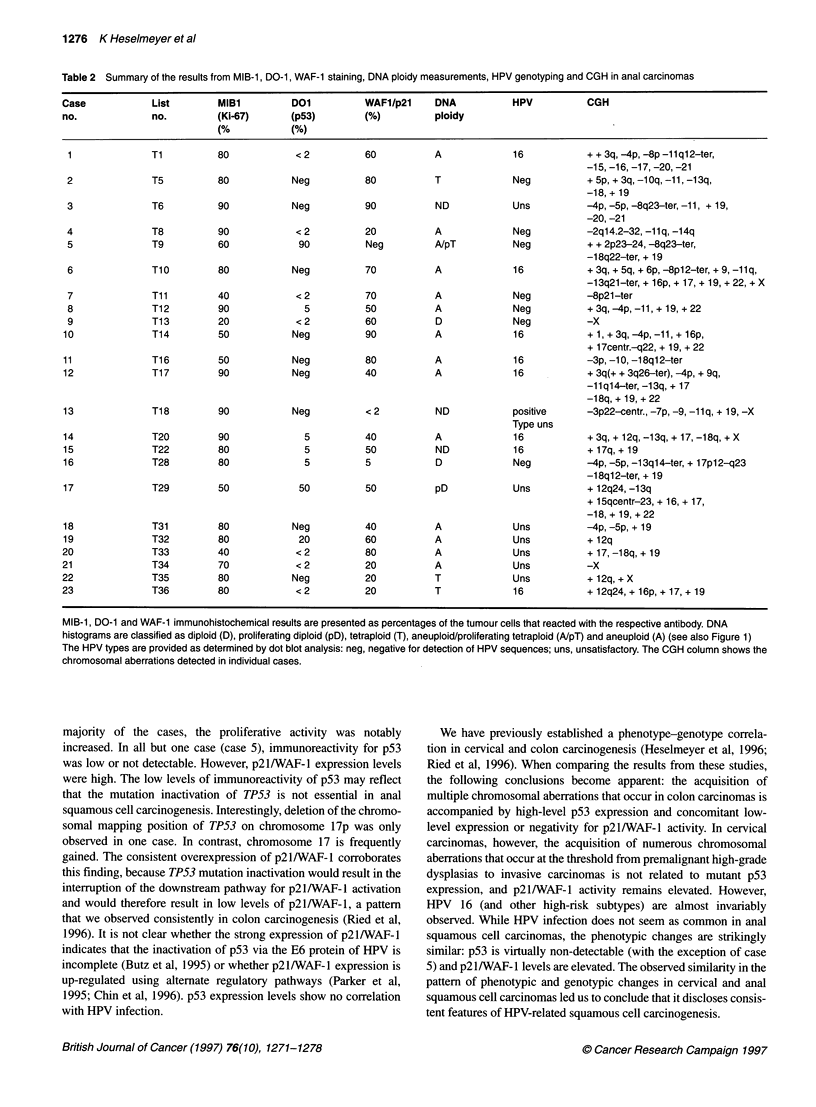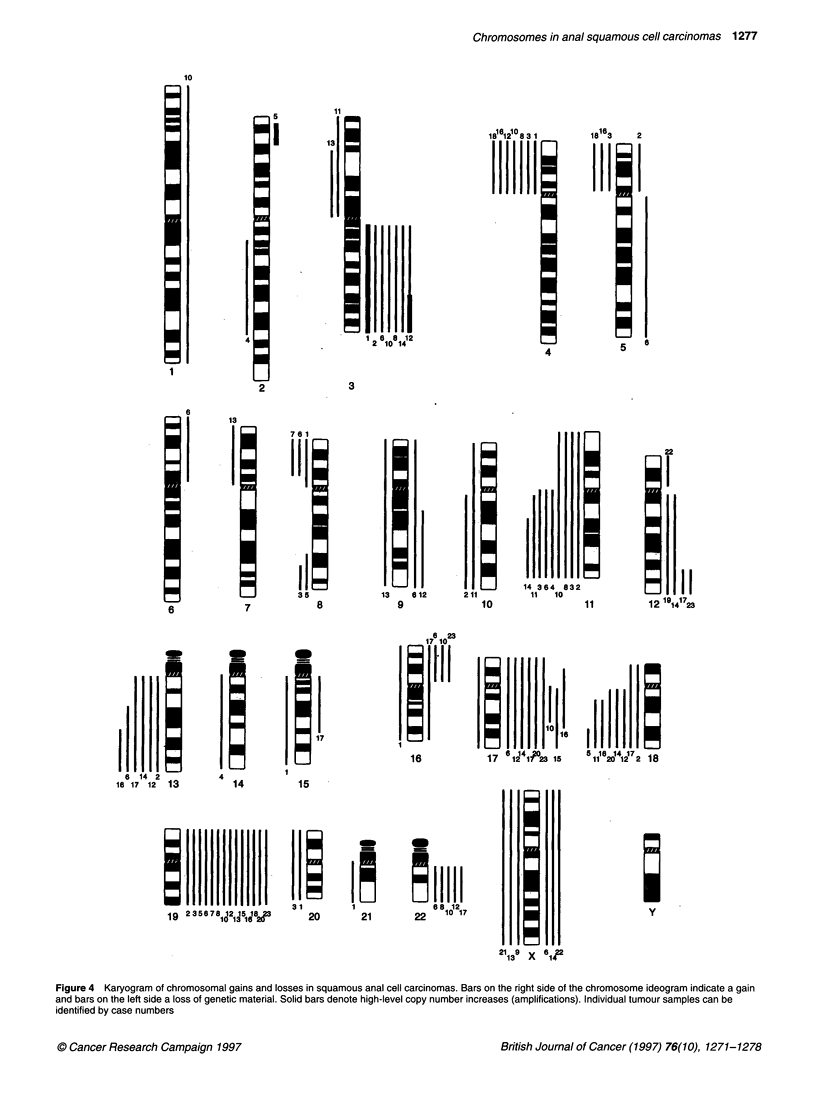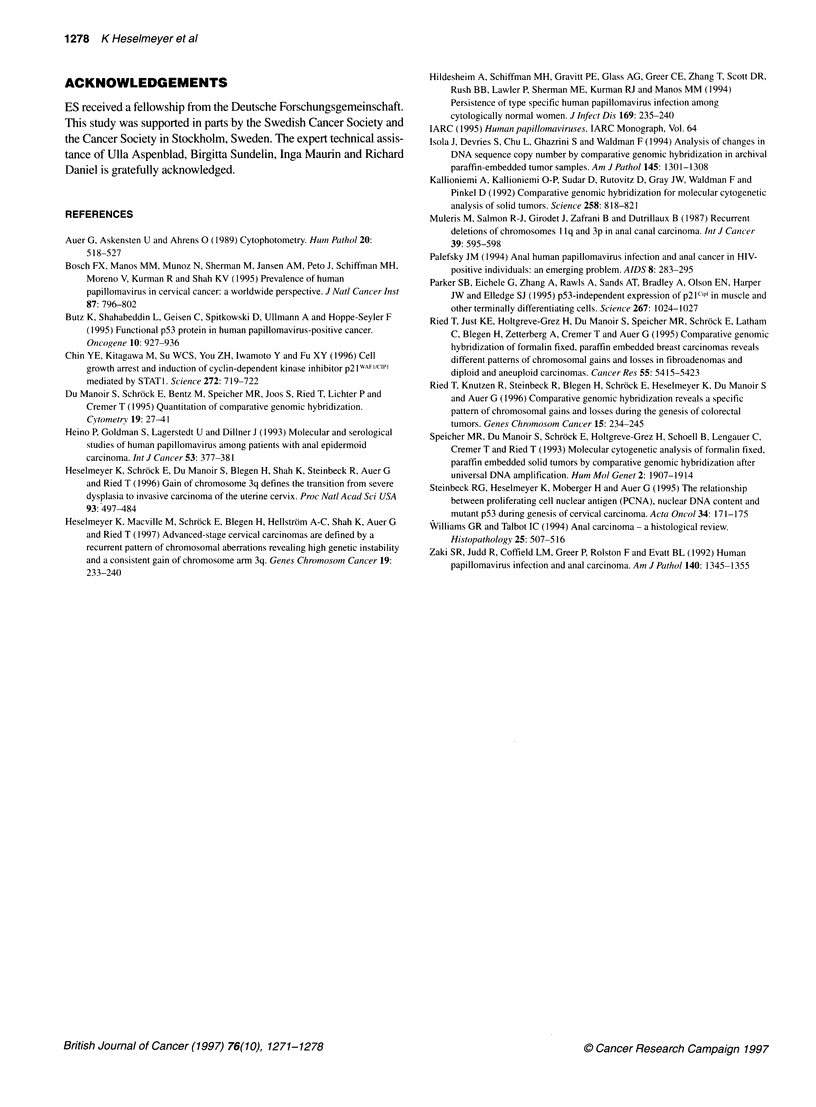# A recurrent pattern of chromosomal aberrations and immunophenotypic appearance defines anal squamous cell carcinomas.

**DOI:** 10.1038/bjc.1997.547

**Published:** 1997

**Authors:** K. Heselmeyer, S. du Manoir, H. Blegen, B. Friberg, C. Svensson, E. SchrÃ¶ck, T. Veldman, K. Shah, G. Auer, T. Ried

**Affiliations:** National Human Genome Research Institute, NIH, Bethesda, MD 20892, USA.

## Abstract

**Images:**


British Journal of Cancer (1997) 76(10), 1271–1278
© 1997 Cancer Research Campaign

### A recurrent pattern of chromosomal aberrations and immunophenotypic appearance defines anal squamous cell carcinomas

**K Heselmeyer[1,2], S du Manoir[1], H Blegen[2], B Friberg[3], C Svensson[4], E Schröck[1], T Veldman[1], K Shah[5], G Auer[2] and T Ried[1]**

[1]National Human Genome Research Institute, NIH, 49 Convent Drive, Building 49/4A28, Bethesda, MD 20892, USA; [2]Division of Cell and Molecular Analysis, Department of Tumor Pathology, Karolinska Institute, Building Z1-01, 01,17176 Stockholm, Sweden; [3]Department of Surgery, Södersjukhuset, Stockholm, Sweden; [4]Huddinge Hospital, Stockholm, Sweden; [5]Johns Hopkins University School of Hygiene and Public Health, 615 North Wolfe Street, Baltimore, MD 21205, USA

**Summary** Squamous cell carcinomas of the anus are rare neoplasias that account for about 3% of large bowel tumours. Infections with human papillomaviruses are frequently detected in these cancers, suggesting that pathogenic pathways in anal carcinomas and in carcinomas of the uterine cervix are similar. Little is known regarding recurrent chromosomal aberrations in this subgroup of squamous cell carcinomas. We have applied comparative genomic hybridization to identify chromosomal gains and losses in 23 cases of anal carcinomas. A non-random copy number increase of chromosomes 17 and 19, and chromosome arm 3q was observed. Consistent losses were mapped to chromosome arms 4p, 11q, 13q and 18q. A majority of the tumours were aneuploid, and most of them showed increased proliferative activity as determined by staining for Ki-67 antigen. p53 expression was low or undetectable, and expression of p21/WAF-1 was increased in most tumours. Sixteen cancers were satisfactorily tested for the presence of HPV by consensus L1-primer polymerase chain reaction; nine were HPV positive, of which eight were positive for HPV 16.

**Keywords:** anal carcinoma; chromosome aberration; immunophenotype; human papillomavirus; comparative genomic hybridization

Comparative genomic hybridization (CGH) serves as a cytogenetic screening test that allows the mapping of chromosomal copy number changes in tumour genomes on normal metaphase chromosomes (Kallioniemi et al; 1992). Only genomic tumour DNA is required for analysis. Consequently, CGH can be performed using DNA extracted from formalin-fixed and paraffin-embedded specimens (Speicher et al, 1993; Isola et al, 1994). This feature allows the retrospective determination of chromosomal aberrations in archived tumour material (Ried et al, 1995). CGH is a well-suited tool to compare chromosomal aberrations with additional pertinent characteristics of specific tumours, such as histology, DNA ploidy and immunohistochemistry (Heselmeyer et al, 1996; Ried et al, 1996). We have recently established a phenotype–genotype correlation in the genesis of HPV-positive squamous cell carcinomas of the cervix uteri (Heselmeyer et al, 1996, 1997). We observed a characteristic pattern of genetic and phenotypic changes that were specific for cervical carcinogenesis: the gain of the long arm of chromosome 3 occurred in virtually all HPV-positive, aneuploid invasive carcinomas; and low expression of p53 was accompanied by high levels of p21/WAF-1. In order to understand whether the observed genotype–phenotype pattern represents a common pathogenic pathway in HPV-induced squamous cell carcinomas, we have extended the CGH analysis to squamous cell carcinomas of the anus. Anal squamous cell carcinomas are rare tumours for which natural history is incompletely understood (Williams et al, 1994). It is clear, however, that HPV genomes are frequently detected in these tumours and their precursor lesions (Zaki et al, 1992; Heino et al, 1993; Palefsky, 1994) and that cervical and anal cancers have similar risk factors and pathology (IARC monograph, 1995). Only a few reports on the cytogenetics of anal carcinomas are available. Muleris et al (1987) identified the recurrent loss of chromosome arms 3p and 11q in a series of eight tumours (Muleris et al, 1987). We used CGH to screen 23 carcinomas of the anus for chromosomal copy number changes. The same tumour section that was used for CGH was analysed for the presence of HPV genomes. Adjacent serial sections were examined for crude DNA ploidy by image cytometry, for proliferative activity (MIB-1), for the expression level of the tumour-suppressor gene *TP53* and the cyclin-dependent kinase inhibitor p21/WAF1.

*Received 3 January 1997*
*Revised 22 April 1997*
*Accepted 30 April 1997*

*Correspondence to:* T Ried

### MATERIALS AND METHODS

### Tissue microdissection and DNA extraction

Tumour material was collected at hospitals throughout Sweden between 1985 and 1989 and diagnosed on haematoxylin and eosin-stained tissue sections. The clinical data are summarized in Table 1. Consecutive sections were prepared from each sample and used for histological diagnosis (4 μm thick), DNA ploidy measurement (8 μm), immunohistochemistry (4 μm) and genomic DNA extraction (50 μm). A second 4-μm section was stained to confirm the presence of tumour tissue. All analyses were performed on these samples.

**Table 1** Summary of the clinical and histomorphological data for 23 anal carcinomas

| Case no. | List no. | Age | Sex | Location | Size (cm) | Stage | Histologic type | Differentiation |
|---|---|---|---|---|---|---|---|---|
| 1 | T1 | 83 | M | Anal | 3 × 2 | pT2, pN0, M0 | Squamous | Poor |
| 2 | T5 | 79 | M | Anal | 4 × 6 | pT3, pN1, M0 | Squamous | Moderate |
| 3 | T6 | 71 | F | Anal | 3 × 1 × 1 | pT2, pN0, M0 | Squamous | Moderate |
| 4 | T8 | 81 | F | Anal | 5 × 8 | pT4, pN1, M1 | Basaloid | High |
| 5 | T9 | 82 | F | Perianal | 2.5 × 1.5 | pT2, pN0, M0 | Basaloid | High |
| 6 | T10 | 70 | M | Anal | 2 × 9 | pT3, pN0, M1 | Basaloid | High |
| 7 | T11 | 74 | F | Anal | 5.5 × 4 | pT3, pN0, M0 | Basaloid | High |
| 8 | T12 | 53 | F | Anal | 4 × 3 | pT2, pN0, M0 | Squamous | High |
| 9 | T13 | 63 | F | Perianal | 2 | pT1, pN0, M0 | Squamous | High |
| 10 | T14 | 63 | F | Anal | 3 × 3 | pT2, pN0, M0 | Basaloid | High |
| 11 | T16 | 83 | F | Anal | 10 × 10 | pT3, pN0, M0 | Squamous | High |
| 12 | T17 | 62 | F | Anal | 3 × 0.5 | pT2, pN0, M0 | Basaloid | High |
| 13 | T18 | 66 | F | Anal | 1 × 1 | pT1, pN0, M0 | Basaloid | High |
| 14 | T20 | 76 | M | Anal | 2 × 1 | pT2, pN0, M0 | Squamous | Moderate |
| 15 | T22 | 37 | F | Perianal | 2 × 1 × 1 | pT1, pN0, M0 | Squamous | High |
| 16 | T28 | 77 | M | Perianal | 2 × 2 | pT2, pN0, M0 | Squamous | High |
| 17 | T29 | 66 | F | Perianal | 1 × 1 | pT1, pN0, M0 | Squamous | High |
| 18 | T31 | 38 | M | Anal | 2 × 3 | pT2, pN0, M0 | Squamous | High |
| 19 | T32 | 67 | F | Anal | 5 × 5 | pT3, pN0, M0 | Squamous | Poor |
| 20 | T33 | 36 | F | Anal | 8 × 4 | pT4, pN1, M0 | Squamous | High |
| 21 | T34 | 52 | F | Anal | 4 | pT2, pN0, M0 | Squamous | Moderate |
| 22 | T35 | 51 | M | Anal | 2.5 × 1.5 | pT2, pN1, M0 | Basaloid | High |
| 23 | T36 | 66 | F | Anal | 4 × 3 | pT2, pN0, M0 | Squamous | High |

### DNA ploidy measurements

DNA content measurements were performed by image cytometry of histological sections as described (Auer et al, 1989; Steinbeck et al, 1995). All DNA values were expressed in relation to the corresponding staining controls, which were given the value 2c, denoting the normal diploid DNA content, and are presented in relative units. The histograms were divided into five groups: (1) diploid cases with a distinct peak in the normal 2c region and no cells or only a minor fraction of cells (< 5%) in the region between 2c and 4c; (2) proliferating diploid cases with a peak in the normal 2c region and a fraction of cells (> 5%) between 2c and 4c; (3) tetraploid cases with a main peak in the 4c region and no cells or only a minor fraction of cells (< 5%) exceeding 5c; (4) aneuploid/proliferating tetraploid cases with a peak in the 4c region and a fraction of 5–20% of the cells exceeding 5c; and (5) aneuploid cases with a main peak around the 4c region and more than 20% of the cells exceeding 5c. Examples of the histograms are presented in Figure 1.

### Immunohistochemistry

Ki-67 antigen was detected with a monoclonal antibody, MIB-1 (Immunotech, Marseille, France). The antibody allows the discrimination of non-proliferating ($G_0$) cells from proliferating cells ($G_1$-S-$G_2$-M). p21/WAF-1 expression was analysed with the WAF-1 antibody EA10 (Oncogene Science), and p53 expression was determined with the DO-1 antibody (Santa Cruz). Immunohistochemistry was performed essentially as described (Ried et al, 1996). Examples of the immunohistochemical staining are presented in Figure 2.

### HPV genotyping

HPV genomes in the purified specimen DNA were identified by polymerase chain reaction (PCR) using the MY09-MY11 L1 consensus primers for amplification (Bosch et al, 1995). Twenty-five type-specific probes and a generic HPV probe were used to diagnose the HPV type in the PCR products as described in detail elsewhere (Hildesheim et al, 1994). β-Globin amplification was performed to evaluate adequacy of tumour DNA.

### CGH

CGH was performed on normal metaphase chromosomes prepared after standard procedures. Control DNA was labelled with digoxigenin dUTP (Boehringer Mannheim) by nick translation. Tumour DNA was extracted from 50-μm formalin-fixed tissue sections. The tissues were dewaxed in xylene and washed in ethanol. The sections were incubated overnight in 1 M sodium-isothiocyanate at 37°C, washed twice in phosphate-suffered saline (PBS) and incubated in DNA extraction buffer (75 mM sodium chloride, 25 mM EDTA, 0.5% Tween). Proteinase K was added to a final concentration of 1 mg $ml^{-1}$. The DNA was phenol extracted and precipitated in ethanol.

Labelling was performed by nick translation substituting dTTP by biotin-16-dUTP (Boehringer Mannheim). Of each differentially labelled genome, 500 ng was precipitated together with an excess (30 μg) of the Cot-1 fraction of human DNA (Gibco BRL). The probe DNA was resuspended in 10 μl of hybridization solution (50% formamide, 2 × standard saline citrate (SSC), 10% dextran sulphate), denatured (5 min, 75°C) and preannealed for 1 h at 37°C. The normal metaphase chromosomes were denatured separately (70% formamide, 2 × SSC) for 2 min at 75°C and dehydrated through an ethanol series. Hybridization took place under a coverslip for 2–4 days at 37°C. Post-hybridization washes and immunocytochemical detection was performed as described (Heselmeyer et al, 1996). Biotin-labelled tumour sequences were detected with avidin conjugated to FITC (Vector laboratories), and the digoxigenin-labelled reference DNA was developed using a mouse anti-digoxigenin antibody, followed by a TRITC-conjugated anti-mouse antibody (Sigma Chemicals). The slides were

© Cancer Research Campaign 1997

**Figure 1** Representative examples of DNA histograms in anal squamous cell carcinomas. The histograms were divided in five groups: (**A**) diploid histograms; (**B**) proliferating diploid; (**C**) tetraploid; (**D**) aneuploid/proliferating tetraploid; and (**E**) aneuploid. For classification of the histograms see Materials and methods counterstained with DAPI and embedded in an antifade solution containing DABCO.

### Microscopy and image analysis

Grey-level images were acquired for each fluorochrome with a cooled CCD camera (Photometrics, Tucson, AZ, USA) coupled to a Leica DMRBE epifluorescence microscope using sequential exposure through fluorochrome specific filters (TR1, TR2, TR3, Chroma Technology). Chromosomes were identified using DAPI-banding. Fluorescence ratio images were calculated by a custom computer program (du Manoir et al, 1995) and run on a Macintosh Quadra 950 (Figure 2D and F). Average ratio profiles were calculated from at least eight ratio images. After the determination of the chromosomal axis for each chromosome in every metaphase, individual FITC and TRITC profiles were calculated. These were used for the computation of the ratio (FITC/TRITC) profiles. The three vertical lines on the right side of the chromosome ideogram represent different values of the fluorescence ratios between the tumour and the normal DNA (Figure 3). The values are 0.75, 1 and 1.25 from left to right. These values were chosen as thresholds for the identification of DNA copy number decreases (below 0.75) and increases (above 1.25) as described in du Manoir et al (1995). The curve shows the ratio profiles that were computed as mean values of at least eight metaphase spreads. Average ratio profiles were the basis for all evaluations.

### RESULTS

CGH was used to identify chromosomal copy number changes in a series of 23 anal squamous cell carcinomas. The clinical data are presented in Table 1. The molecular cytogenetic analysis was complemented by HPV genotyping and DNA ploidy measurements. The proliferative activity and the expression levels of the tumour-suppressor gene *p53* and the cyclin-dependent kinase inhibitor p21/WAF-1 were analysed on the same tissue sections. The combined data allow for a phenotype/genotype correlation in this rare subgroup of squamous cell carcinomas. Table 2 summarizes the data.

### CGH

The CGH analysis revealed a recurrent pattern of chromosomal gains and losses. Figure 2D and F presents ratio images from tumours 5 and 8. An average ratio profile for case 8 is presented in Figure 3. The average ratio profile was used for the identification of copy number changes in all cases. The karyogram of chromosomal gains and losses is summarized in Figure 4. Chromosomal gains were mapped to chromosome 19 (14 of 23 cases), chromosome 17 (9 of 23 cases), chromosome 3q (7 of 23 cases) and chromosome 22 (5 of 23 cases). Consistent losses occurred on chromosomes 11 (9 of 23 cases), 18 (8 of 23 cases), 4p (7 of 23 cases) and 13 (6 of 23 cases). High-level copy number increases were observed infrequently on chromosome 3q (two cases) and on chromosome band 2p23–24. All tumours showed chromosomal copy number alterations. In 23 tumours, 110 copy number changes were observed, resulting in an average number of copy alterations (ANCA) of 4.8. In 90 of 110 copy number changes (82%), entire chromosomes or chromosome arms were subject to gain or loss.

**Figure 2** Examples of immunohistochemical detection of the Ki-67 antigen, p53 and p21/WAF-1 expression in case 8 (**A–C**) and 5 (**E–G**). Immunoreactive nuclei appear dark brown. The tissue was counterstained with haematoxylin and eosin, which appears blue. The CGH ratio images of these cases are displayed in **D** and **H**. Blue indicates a balance between tumour and test genomes, red reflects a loss of genetic material in the tumour DNA and green shows regions that are gained in the tumour. Case 5 reveals increased proliferative activity, intense staining with the DO-1 antibody and low reactivity with the antibody against p21/WAF-1. Case 8, however, revealed a pattern that was recurrently observed in anal cell carcinomas, i.e. increased proliferative activity, undetectable p53 staining and high expression of p21/WAF-1

*© Cancer Research Campaign 1997*

**Figure 3** Identification of copy number changes by CGH. Average ratio profile of case 8. The three vertical lines on the right side of the chromosome ideograms reflect different values of the fluorescence ratio between the tumor and the normal DNA. The values are 0.75, 1 and 1.25 from left to right. The ratio profile (curve) was calculated as a mean value of at least eight metaphase spreads. Copy number changes are present on chromosome 3q,19 and 22 (gains) and 4p and 11 (losses)

### HPV genotyping

HPV genotyping was performed on the genomic tumour DNA that was used for CGH as described. Seven tumour DNAs in which β-globin could not be amplified were scored as unsatisfactory. In the remaining sixteen tumours, HPV 16 was detected in eight tumours; in one tumour, the HPV type could not be identified. Seven tumours were negative for HPV. Aneuploid DNA histograms were seen in six of seven HPV-positive and four of seven HPV-negative tumours. The data are summarized in Table 2.

### DNA ploidy

The majority of the cases studied by image cytometry on consecutive sections revealed an aneuploid DNA histogram with a main peak in the tetraploid region (4c) and more than 20% of the cells exceeding 5c. Only two of the anal carcinomas remained diploid. One of the diploid tumours revealed, as a sole anomaly, the loss of one X chromosome. However, the second diploid tumour showed multiple chromosomal aberrations. Three tumours revealed a clear tetraploidization with peak values between 3.9c and 4.1c. There was no obvious correlation between the number of copy alterations and the ploidy. Examples of the histograms are presented in Figure 1 and a summary of the ploidy measurements is given in Table 2.

### Immunohistochemistry

Proliferative activity and p53 and p21/WAF-1 expression levels were investigated immunohistochemically. Seventeen of 23 tumours revealed increased proliferative activity, with more than 60% of the cells showing positive staining with an antibody against Ki-67. Increased immunoreactivity for p53 was low or not detectable with the exception of one case, in which 90% of the cells showed immunoreactivity (case 5). The percentage of the cells that stained positively with an antibody directed against p21/WAF-1 ranged from 20% to 90%, with most of the cases showing moderately to highly increased reactivity in 40–60% of the cells. Immunoreactivity was not detected in one case (case 5) in which p53 expression was high, suggesting the presence of mutated *TP53*. Figure 2 presents examples of the immunohistochemistry of case 8, which exemplifies the consistent staining pattern, and of case 5, which revealed an exceptional immunostaining. A summary of the quantification is provided in Table 2.

### DISCUSSION

We conducted a molecular cytogenetic study of 23 cases of anal squamous cell carcinomas by CGH. The cytogenetic data were complemented by DNA ploidy measurements, HPV genotyping and expression level analysis of the proliferation marker Ki-67, the tumour-suppressor gene product *p53* and the cyclin-dependent kinase inhibitor p21/WAF-1. CGH analysis revealed a recurrent pattern of chromosomal gains and losses. Chromosomes 11, 18, 13 and 4p were most frequently under-represented, whereas copy number gains were consistently mapped to chromosomes 19, 17 and 3q. It should be noted that copy number changes involving chromosome 19 require cautious interpretation if they occur simultaneously with changes on chromosomes 1p, 16 and 22. These chromosomal regions are known to be rich in Alu-repeat sequences, which may cause unspecific ratio variations. However, chromosome 19 was independently subjected to copy number increases. In three instances, high-level copy number increases (amplifications) were identified on chromosome arm 3q and or chromosomal bands 3q26–28 and 2p23–24. The amplification on 2p24 coincides with the chromosomal mapping position of the N-myc proto-oncogene. Reports on chromosomal aberrations by means of chromosome banding analyses are rare. Muleris et al (1987) reported the non-random loss of chromosomes 3p and 11q. While chromosome 3p is affected in only 2 of 23 cases in our study, the loss of chromosome 11q occurs in 9 of 23 of our cases. The minimally deleted region encompasses chromosome bands 11q14–25. The recurrent loss of this chromosomal region suggests that this region harbours a tumour-suppressor gene involved in the genesis of anal squamous cell carcinomas. The average number of copy alterations (ANCA) amounts to 4.8 per tumour. In a previously performed series of stage T1 cervical carcinomas, the ANCA per tumour was 4.0 (Heselmeyer et al, 1996). No correlation between the ANCA per tumour and the ploidy, the presence of HPV infection or the differentiation and tumour stage was obvious in anal squamous cell carcinomas. The immunohistochemical analysis of the same samples that were used for CGH revealed a consistent pattern: in the

© Cancer Research Campaign 1997 

Table 2 Summary of the results from MIB-1, DO-1, WAF-1 staining, DNA ploidy measurements, HPV genotyping and CGH in anal carcinomas

| Case no. | List no. | MIB1 (Ki-67) (% | DO1 (p53) (%) | WAF1/p21 (%) | DNA ploidy | HPV | CGH |
|---|---|---|---|---|---|---|---|
| 1 | T1 | 80 | < 2 | 60 | A | 16 | + + 3q, –4p, –8p –11q12–ter, –15, –16, –17, –20, –21 |
| 2 | T5 | 80 | Neg | 80 | T | Neg | + 5p, + 3q, –10q, –11, –13q, –18, + 19 |
| 3 | T6 | 90 | Neg | 90 | ND | Uns | –4p, –5p, –8q23–ter, –11, + 19, –20, –21 |
| 4 | T8 | 90 | < 2 | 20 | A | Neg | –2q14.2–32, –11q, –14q |
| 5 | T9 | 60 | 90 | Neg | A/pT | Neg | + + 2p23–24, –8q23–ter, –18q22–ter, + 19 |
| 6 | T10 | 80 | Neg | 70 | A | 16 | + 3q, + 5q, + 6p, –8p12–ter, + 9, –11q, –13q21–ter, + 16p, + 17, + 19, + 22, + X |
| 7 | T11 | 40 | < 2 | 70 | A | Neg | –8p21–ter |
| 8 | T12 | 90 | 5 | 50 | A | Neg | + 3q, –4p, –11, + 19, + 22 |
| 9 | T13 | 20 | < 2 | 60 | D | Neg | –X |
| 10 | T14 | 50 | Neg | 90 | A | 16 | + 1, + 3q, –4p, –11, + 16p, + 17centr.–q22, + 19, + 22 |
| 11 | T16 | 50 | Neg | 80 | A | 16 | –3p, –10, –18q12–ter |
| 12 | T17 | 90 | Neg | 40 | A | 16 | + 3q(+ + 3q26–ter), –4p, + 9q, –11q14–ter, –13q, + 17 –18q, + 19, + 22 |
| 13 | T18 | 90 | Neg | < 2 | ND | positive Type uns | –3p22–centr., –7p, –9, –11q, + 19, –X |
| 14 | T20 | 90 | 5 | 40 | A | 16 | + 3q, + 12q, –13q, + 17, –18q, + X |
| 15 | T22 | 80 | 5 | 50 | ND | 16 | + 17q, + 19 |
| 16 | T28 | 80 | 5 | 5 | D | Neg | –4p, –5p, –13q14–ter, + 17p12–q23 –18q12–ter, + 19 |
| 17 | T29 | 50 | 50 | 50 | pD | Uns | + 12q24, –13q + 15qcentr–23, + 16, + 17, –18, + 19, + 22 |
| 18 | T31 | 80 | Neg | 40 | A | Uns | –4p, –5p, + 19 |
| 19 | T32 | 80 | 20 | 60 | A | Uns | + 12q |
| 20 | T33 | 40 | < 2 | 80 | A | Uns | + 17, –18q, + 19 |
| 21 | T34 | 70 | < 2 | 20 | A | Uns | –X |
| 22 | T35 | 80 | Neg | 20 | T | Uns | + 12q, + X |
| 23 | T36 | 80 | < 2 | 20 | T | 16 | + 12q24, + 16p, + 17, + 19 |

MIB-1, DO-1 and WAF-1 immunohistochemical results are presented as percentages of the tumour cells that reacted with the respective antibody. DNA histograms are classified as diploid (D), proliferating diploid (pD), tetraploid (T), aneuploid/proliferating tetraploid (A/pT) and aneuploid (A) (see also Figure 1) The HPV types are provided as determined by dot blot analysis: neg, negative for detection of HPV sequences; uns, unsatisfactory. The CGH column shows the chromosomal aberrations detected in individual cases.

majority of the cases, the proliferative activity was notably increased. In all but one case (case 5), immunoreactivity for p53 was low or not detectable. However, p21/WAF-1 expression levels were high. The low levels of immunoreactivity of p53 may reflect that the mutation inactivation of *TP53* is not essential in anal squamous cell carcinogenesis. Interestingly, deletion of the chromosomal mapping position of *TP53* on chromosome 17p was only observed in one case. In contrast, chromosome 17 is frequently gained. The consistent overexpression of p21/WAF-1 corroborates this finding, because *TP53* mutation inactivation would result in the interruption of the downstream pathway for p21/WAF-1 activation and would therefore result in low levels of p21/WAF-1, a pattern that we observed consistently in colon carcinogenesis (Ried et al, 1996). It is not clear whether the strong expression of p21/WAF-1 indicates that the inactivation of p53 via the E6 protein of HPV is incomplete (Butz et al, 1995) or whether p21/WAF-1 expression is up-regulated using alternate regulatory pathways (Parker et al, 1995; Chin et al, 1996). p53 expression levels show no correlation with HPV infection.

We have previously established a phenotype–genotype correlation in cervical and colon carcinogenesis (Heselmeyer et al, 1996; Ried et al, 1996). When comparing the results from these studies, the following conclusions become apparent: the acquisition of multiple chromosomal aberrations that occur in colon carcinomas is accompanied by high-level p53 expression and concomitant low-level expression or negativity for p21/WAF-1 activity. In cervical carcinomas, however, the acquisition of numerous chromosomal aberrations that occur at the threshold from premalignant high-grade dysplasias to invasive carcinomas is not related to mutant p53 expression, and p21/WAF-1 activity remains elevated. However, HPV 16 (and other high-risk subtypes) are almost invariably observed. While HPV infection does not seem as common in anal squamous cell carcinomas, the phenotypic changes are strikingly similar: p53 is virtually non-detectable (with the exception of case 5) and p21/WAF-1 levels are elevated. The observed similarity in the pattern of phenotypic and genotypic changes in cervical and anal squamous cell carcinomas led us to conclude that it discloses consistent features of HPV-related squamous cell carcinogenesis.

© Cancer Research Campaign 1997

**Figure 4** Karyogram of chromosomal gains and losses in squamous anal cell carcinomas. Bars on the right side of the chromosome ideogram indicate a gain and bars on the left side a loss of genetic material. Solid bars denote high-level copy number increases (amplifications). Individual tumour samples can be identified by case numbers

© Cancer Research Campaign 1997

### ACKNOWLEDGEMENTS

ES received a fellowship from the Deutsche Forschungsgemeinschaft. This study was supported in parts by the Swedish Cancer Society and the Cancer Society in Stockholm, Sweden. The expert technical assistance of Ulla Aspenblad, Birgitta Sundelin, Inga Maurin and Richard Daniel is gratefully acknowledged.

### REFERENCES

Auer G, Askensten U and Ahrens O (1989) Cytophotometry. *Hum Pathol* **20**: 518–527

Bosch FX, Manos MM, Munoz N, Sherman M, Jansen AM, Peto J, Schiffman MH, Moreno V, Kurman R and Shah KV (1995) Prevalence of human papillomavirus in cervical cancer: a worldwide perspective. *J Natl Cancer Inst* **87**: 796–802

Butz K, Shahabeddin L, Geisen C, Spitkowski D, Ullmann A and Hoppe-Seyler F (1995) Functional p53 protein in human papillomavirus-positive cancer. *Oncogene* **10**: 927–936

Chin YE, Kitagawa M, Su WCS, You ZH, Iwamoto Y and Fu XY (1996) Cell growth arrest and induction of cyclin-dependent kinase inhibitor $p21^{WAF1/CIP1}$ mediated by STAT1. *Science* **272**: 719–722

Du Manoir S, Schröck E, Bentz M, Speicher MR, Joos S, Ried T, Lichter P and Cremer T (1995) Quantitation of comparative genomic hybridization. *Cytometry* **19**: 27–41

Heino P, Goldman S, Lagerstedt U and Dillner J (1993) Molecular and serological studies of human papillomavirus among patients with anal epidermoid carcinoma. *Int J Cancer* **53**: 377–381

Heselmeyer K, Schröck E, Du Manoir S, Blegen H, Shah K, Steinbeck R, Auer G and Ried T (1996) Gain of chromosome 3q defines the transition from severe dysplasia to invasive carcinoma of the uterine cervix. *Proc Natl Acad Sci USA* **93**: 497–484

Heselmeyer K, Macville M, Schröck E, Blegen H, Hellström A-C, Shah K, Auer G and Ried T (1997) Advanced-stage cervical carcinomas are defined by a recurrent pattern of chromosomal aberrations revealing high genetic instability and a consistent gain of chromosome arm 3q. *Genes Chromosom Cancer* **19**: 233–240

Hildesheim A, Schiffman MH, Gravitt PE, Glass AG, Greer CE, Zhang T, Scott DR, Rush BB, Lawler P, Sherman ME, Kurman RJ and Manos MM (1994) Persistence of type specific human papillomavirus infection among cytologically normal women. *J Infect Dis* **169**: 235–240

IARC (1995) *Human papillomaviruses*. IARC Monograph, Vol. 64

Isola J, Devries S, Chu L, Ghazrini S and Waldman F (1994) Analysis of changes in DNA sequence copy number by comparative genomic hybridization in archival paraffin-embedded tumor samples. *Am J Pathol* **145**: 1301–1308

Kallioniemi A, Kallioniemi O-P, Sudar D, Rutovitz D, Gray JW, Waldman F and Pinkel D (1992) Comparative genomic hybridization for molecular cytogenetic analysis of solid tumors. *Science* **258**: 818–821

Muleris M, Salmon R-J, Girodet J, Zafrani B and Dutrillaux B (1987) Recurrent deletions of chromosomes 11q and 3p in anal canal carcinoma. *Int J Cancer* **39**: 595–598

Palefsky JM (1994) Anal human papillomavirus infection and anal cancer in HIV-positive individuals: an emerging problem. *AIDS* **8**: 283–295

Parker SB, Eichele G, Zhang A, Rawls A, Sands AT, Bradley A, Olson EN, Harper JW and Elledge SJ (1995) p53-independent expression of $p21^{Cip1}$ in muscle and other terminally differentiating cells. *Science* **267**: 1024–1027

Ried T, Just KE, Holtgreve-Grez H, Du Manoir S, Speicher MR, Schröck E, Latham C, Blegen H, Zetterberg A, Cremer T and Auer G (1995) Comparative genomic hybridization of formalin fixed, paraffin embedded breast carcinomas reveals different patterns of chromosomal gains and losses in fibroadenomas and diploid and aneuploid carcinomas. *Cancer Res* **55**: 5415–5423

Ried T, Knutzen R, Steinbeck R, Blegen H, Schröck E, Heselmeyer K, Du Manoir S and Auer G (1996) Comparative genomic hybridization reveals a specific pattern of chromosomal gains and losses during the genesis of colorectal tumors. *Genes Chromosom Cancer* **15**: 234–245

Speicher MR, Du Manoir S, Schröck E, Holtgreve-Grez H, Schoell B, Lengauer C, Cremer T and Ried T (1993) Molecular cytogenetic analysis of formalin fixed, paraffin embedded solid tumors by comparative genomic hybridization after universal DNA amplification. *Hum Mol Genet* **2**: 1907–1914

Steinbeck RG, Heselmeyer K, Moberger H and Auer G (1995) The relationship between proliferating cell nuclear antigen (PCNA), nuclear DNA content and mutant p53 during genesis of cervical carcinoma. *Acta Oncol* **34**: 171–175

Williams GR and Talbot IC (1994) Anal carcinoma – a histological review. *Histopathology* **25**: 507–516

Zaki SR, Judd R, Coffield LM, Greer P, Rolston F and Evatt BL (1992) Human papillomavirus infection and anal carcinoma. *Am J Pathol* **140**: 1345–1355

© Cancer Research Campaign 1997